# Education, Class, and Female Genital Cutting among the Samburu of Northern Kenya: Challenging the Reproduction of the “Ignorant Pastoralist” Narrative in Anticutting Campaigns

**DOI:** 10.1177/10778012221079376

**Published:** 2022-04-14

**Authors:** Hannelore Van Bavel

**Affiliations:** Anthropology & Sociology, 4913 SOAS University of London, London, UK

**Keywords:** female genital mutilation/cutting, pastoralists, formal education, class, Samburu

## Abstract

Based on ethnographic research among the Samburu of northern Kenya, this article examines the association between formal education and the abandonment of female genital mutilation/cutting (FGM/C). It challenges the notion that Samburu continue cutting out of “ignorance” of the health and legal implications of cutting. The findings show that, rather than a causal effect of “knowledge” on cutting-related attitudes and behavior, formal education can replace FGM/C as a source for status, respect, and adulthood. In addition, alternative expectations apply to formally educated Samburu. Challenging the reproduction of the “ignorant pastoralist” narrative in anticutting campaigns is important because of the harm such narratives inflict on pastoralist communities.

## Introduction

The World Health Organization defines female genital mutilation/cutting (FGM/C) as the partial or total removal of external female genitalia or other injury to the female genital organs for nonmedical reasons (World Health Organization, 2018). FGM/C occurs worldwide, with highly concentrated prevalence in 27 countries in sub-Saharan Africa, Iraq, Yemen, and Indonesia and among diaspora communities in Europe and the United States (UNICEF, 2013). Efforts to end FGM/C date back to the turn of the 20th century, when colonial officials and missionaries in colonial Kenya described the practice as “barbaric” and “primitive,” and tried to stamp it out through law, education, and exclusion from church membership and privileges (Anderson, 2018; Njambi, 2007; Pedersen, 1991; Thomas, 2003). International efforts have intensified since the 1990s, when FGM/C became first recognized as a human rights violation during the 1993 Vienna World Conference on Human Rights (Shell-Duncan, 2008). International commitment to end the practice was reinforced and strengthened by the Resolution to eradicate FGM/C of the General Assembly in 2012 and by the Sustainable Development Goals which include a target to eliminate all harmful practices such as child, early, and forced marriage and FGM/C by 2030 (SDG, 2016; UN General Assembly, 2012).

Kenya is often cited as a model country for its fight against FGM/C; the national prevalence of FGM/C has steadily decreased from 32% in 2003 to 21% in 2014 (KDHS, 2014). In 2011, Kenya passed the Prohibition of Female Genital Mutilation Act, criminalizing not only performing the cut but also possessing the tools for cutting, providing a space for the cut to take place, the use of derogatory language in relation to uncircumcised girls and their partners, and failure to report FGM (Kenya, 2011). Kenya also has a strong civil society presence, consisting of Kenyan and international NGOs and activists using a range of different approaches to ending FGM/C, including community sensitization and dialogue, so-called “alternative rites of passage,” rescue camps for girls who ran away from FGM/C and/or child marriage, and the involvement of religious and cultural leaders.

Besides the influence of anti-FGM legislation and NGO efforts, evidence indicates that formal education has an important influence on whether people support and practice FGM/C (Alo & Gbadebo, 2011; Asekun-Olarinmoye & Amusan, 2008; Gage & Van Rossem, 2006; UNICEF, 2016). In Kenya, the prevalence of FGM/C is 13% for girls whose mothers did not have any formal education in comparison to 1% for girls whose mothers completed primary education (KDHS, 2014). Similarly, a Kenyan woman's likelihood of being cut decreases with an increase in her own educational level (Achia, 2014). A 2017 survey conducted by UNICEF among Rendille, Maasai, Pokot, Samburu, and Somali communities in Kenya found that the “intent to have FGM/C performed on uncut daughters decreases with increasing level of education” (UNICEF, 2017).

However, there is a lack of in-depth understanding of the linkages between formal education and FGM/C to determine whether it is education alone, specific education on health and legal risks of FGM, or other confounding variables that contribute to these changing views (ICRW, 2016, p. 12). This article contributes to understanding the association between formal education and the abandonment of FGM/C by studying ongoing changes in FGM/C among the Samburu of northern Kenya. The findings show that the lower prevalence of FGM/C among formally educated Samburu cannot be explained by higher rates of knowledge on FGM/C and its health, human rights, and other implications: Samburu without formal education showed similar levels of knowledge. Formally educated Samburu more often opposed FGM/C, but so did Samburu with a higher socioeconomic status and Samburu who lived closer to urban centers. These Samburu demonstrated aspirations for a “modern” lifestyle that does not include FGM/C, whereas Samburu without formal education, with lower socioeconomic status, and living in more rural areas more often aspired to upholding “traditions,” including FGM/C. Importantly, whereas some “traditional” Samburu did oppose FGM/C, they found it harder to effectively abandon the practice because of the social sanctions that would follow, such as loss of status and ostracism. Samburu with formal education, a higher socioeconomic status, and/or living closer to town found it easier to avoid or circumvent these sanctions.

Building on these findings, the article interrogates the assumption, sometimes implicitly present in anticutting projects in and beyond Samburu, that people continue cutting out of “ignorance” of the health and legal implications of cutting, that “raising awareness” on these issues will change people's behavior, and, if not, that this reflects the stubborn and backward nature of pastoralist communities like the Samburu. Anthropologists and anticolonial scholars have long criticized anti-FGM discourses for being patronizing and for portraying African women as the passive victims of patriarchal and backward cultures (Mohanty, 1988; Nnaemeka, 2005). Adding to these critiques, I argue that an anti-FGM/C rhetoric that blames pastoralists’ “ignorance” for the high prevalence of FGM/C risks continuing and reinforcing tropes of backward and stubborn pastoralists that date back to colonial times and which, as has been argued by Straight (2020) and others, ultimately legitimize policies that further infantilize and marginalize pastoralists in favor of farmers and Kenyan elites.

## Methodology

This study is part of a nodal ethnography (Hodgson, 2011, p. 18) on the production and travel of discourses on FGM/C (Van Bavel, 2020). I conducted fieldwork in different parts of Kenya (Nairobi, Narok County, Samburu County) between October 2017 and December 2018. In Samburu county, data collection took place in Wamba and Archer's Post (Samburu East), Maralal town and a neighboring village (Samburu West), and Baragoi (Samburu North) in February, September, and October 2018, where the majority of the population identifies as Samburu, an ethnic group of seminomadic pastoralists that is closely related to the Maasai in terms of culture and language (Spencer, 1965).

Fieldwork consisted of participant observation, informal conversations, and 32 in-depth interviews with community members, hospital staff, a police officer, and NGO staff. The inclusion criteria for the in-depth interviews were self-identifying as Samburu and being 16 years or older, or not self-identifying as Samburu but working as a nurse, development staff, or police among the Samburu community. Hospital staff, development agencies staff, and the police officer were asked questions relevant to their professional positions to get an understanding of the health-related, developmental, and legal context of FGM/C in Samburu. The sample of in-depth interviews included more women (24 women vs. 8 men) to balance out the overrepresentation of male voices that I recorded during my participant observation.

Although informal conversations during the phase of participant observation took place in a range of settings and in the presence or absence of others, I made sure that in-depth interviews were carried out in private spaces where participants felt comfortable (such as the participant's home or in an empty office space) without the presence of other people except myself and, when necessary, an interpreter. I conducted interviews in English or Swahili when possible; an interpreter of the same gender as the participants assisted interviews with individuals who spoke Lmaa, the language spoken by Samburu.

My study was approved by the SOAS Ethics Committee and the Kenyan National Commission for Science, Technology and Innovation. I reported to the County Commissioner of Samburu County before starting my research project. Before every interview, I told participants that the purpose of my study was to understand FGM/C, changes that have occurred related to FGM/C, and factors that have contributed to such changes. I explained to the participants that their participation in the study was fully voluntary, and that the data collected would be kept strictly confidential and used for this research's purposes only. Informal conservations during the participant observation phase of this study were recorded in field notes. Although most people were aware of my role as a researcher, I repeated the purpose of my study and asked for explicit oral consent to use elements from our informal conversation anonymously. Five participants were 16 years old at the time of the interview. In these cases, I sought the consent of a parent or guardian in addition to the consent of the minor. In-depth interviews were audio-recorded after obtaining fully informed oral consent and transcribed verbatim. Interviews lasted one hour on average. To ensure confidentially, no names were audio-recorded nor written down in the field notes or interview transcripts. Tape recordings and transcripts were password protected, and access was limited to the researcher.

## Female Genital Mutilation/Cutting among the Samburu

The Samburu are Maa-speaking pastoralists living in Samburu County in northern Kenya, with Maralal as its administrative center. Samburu society is characterized by its livestock economy based on the keeping of cattle, small stock, and camels, supplemented by wage employment, trade, or agriculture. As with other pastoralist societies in East Africa, Samburu people have experienced increasing pressure on their livelihoods, largely because of colonial and post-Independence land alienation for European settlement, national parks, and private ranches. Among these alienated lands are grazing lands crucial for cattle survival during dry seasons. The result has been that Samburu communities have been pressured into diversifying their economy and, at times, accepting foreign food relief (Straight, 2007, p. 26). Pavitt (2006, p. 19) writes that development projects targeting Samburu people have often failed or even backfired because outside “experts” lacked an understanding of Samburu society and did not involve Samburu people.

Samburu society is organized by the age-set principle (Fratkin, 2011, p. 9; Spencer, 1965). Age sets refer to groups of men who have been circumcised during the same interval of roughly 14 years. Circumcision initiates boys into warriorhood, a period during which the initiated warriors are expected to protect the community. Women do not have age sets, but are similarly initiated from girlhood to womanhood through “circumcision,” reaching full status when they birth children (Fratkin, 2011, p. 10). Both male and female circumcision (*muratare*) are thus part of rite of passage ceremonies that initiate boys and girls into adulthood and therefore take place around the onset of puberty. Circumcision raises the status of the initiate and their extended family and is therefore very much part of the social fabric of society. Although male circumcision takes place during annual circumcision periods, women can be cut throughout the year. Usually, a girl is cut on her wedding day, after which she moves into the house of her mother-in-law to heal. Girls who by the time they reach their late teens are still not engaged to be married can be cut without the immediate prospect of marriage. The type of cut most common among Samburu is excision or type II FGM/C (UNICEF, 2017, p. 67), which involves the removal of the protruding part of the clitoris and the labia minora. Interlocutors described “female circumcision” as an important part of the culture that is largely taken for granted: “Every Samburu is circumcised. It is not even something we think about: you just do it because your parents have done it, and their parents have done it, and everyone in the community does it” (female, 28 years old, higher education, unmarried, Maralal).

Circumcision is a prerequisite for marriage and turns boys and girls into full members of the community. Uncircumcised boys and girls are considered children, regardless of their age or physical maturity, and therefore are not allowed to marry or have children. Remaining uncircumcised also affects the status of family members. An older woman, grandmother to young men of circumcision age, explained that her own circumcision affects the status of her children and grandchildren: “The time that we start circumcising our young men, we all go to the lorora^1^ together. But if you, as his mother, haven’t been circumcised yourself, you are not allowed to go circumcise your son. […] your son will never grow up to be a man” (female, ± 75 years old, no formal education, married, Baragoi).

Other interlocutors said that the children of an uncircumcised woman are not acknowledged, because the woman is considered a child herself and “children cannot have children” (female, 70 years old, no formal education, married, village close to Maralal). A key informant emphasized the pressure on families and individuals to conform to societal expectations:

[Outsiders] don’t understand what the consequences are of not practicing. Living in a community like Samburu means you are mutually dependent on the rest of the community. Let’s say you are the one who has many cows, and you are my neighbor, but I don’t have any—you can share your cows with me, so I can milk them for my family. This means that you will influence what I do because I depend on you. So this compromises me; even if I don’t want to practice female circumcision myself, I will still practice it because I fear that if I don’t, I lose my bond with you. (key informant, male, 25 years old, higher education, unmarried, Maralal)

## Changes in FGM/C among the Samburu

Despite the sociocultural importance Samburu attach to FGM/C, the prevalence of FGM/C among the Samburu is decreasing. Whereas in 2014, the FGM/C prevalence in Samburu was estimated at 85% (KDHS, 2014), data collected between 2016 and 2017 suggest a prevalence of 74% (UNICEF, 2017, p. 28). Interlocutors all reported that the practice has been changing over the last decade, with some people abandoning the cut, opting for a less severe type of cutting, or cutting in secret, especially around the urban center of Maralal (Samburu Central) and, to a lesser extent, in rural areas and in Samburu East and North.

Interlocutors mentioned that these changes are not taking place equally across the community. Interlocutors agreed that it was mainly “learned” Samburu and those living in or close to urban centers who abandoned cutting. Some referred to formally educated, urban, and economically well-off Samburu as having adopted a “digital” lifestyle in contrast to the “analogue” lifestyle of rural Samburu without formal education.

Not circumcising girls is something that has come with those digital. [Interviewer: What do you mean by “digital”?] Those from town and from school, with their cars and Swahili^2^ clothes. (female, 18 years old, no formal education, married, village in Samburu West)

The term was used most by rural Samburu without formal education to jokingly refer to Samburu who they perceived as different from themselves and, in particular, as less “traditional” (*lkereti*). When I mentioned the word to my highly educated friend who lives in Maralal, he explained:

That is how people in the village call us. It is a bit like saying we are modern Samburu and they are traditional Samburu. It can mean you have gone to school, but they will even use this for people who have been in Nairobi or own a car or wear jeans—you know, all these things that we don’t have in the village. […] Even Leshan^3^, he is from the village, he didn’t go to college, but he has his bodaboda [motorcycle taxi] business in town, so he is “digital.”

Some interlocutors used the word “*kisasa*” (Swahili for “modern”) to refer to the same people that others referred to as “digital.” Samburu from the more rural areas, with no formal education and lower socioeconomic status, referred to themselves and each other as “traditional” or, jokingly, “analogue.” Although anthropology has long challenged the divide between “tradition” and “modernity,” my interlocutors used these terms to categorize people and explain how FGM/C might be changing among “modern/digital” Samburu, but less among “traditional/analogue” Samburu. In this article, I therefore follow these emic categories. These categories were not clearly delineated but, in general, people categorized someone else as “digital” or “modern” if this person had one or more characteristics of “middle-classness” as described by Spronk (2014, p. 99): “(1) access to education and the resulting salaried occupations, (2) consumption patterns and lifestyle choices, and (3) modern self-perceptions.” Digital/modern Samburu can thus be people who have enjoyed some level of formal education and/or make a modest living from employment in (eco)tourism, conservancy, government jobs, or the NGO sector (whether or not in combination with pastoralism or agriculture) and/or lived or aspired to a “modern” lifestyle. My friend gave the example of Leshan, who did not complete secondary education but operates a motorcycle taxi business, lives close to Maralal, and aspires for his children to do well in school and become lawyers or doctors. Samburu interlocutors with formal education were often younger and more often identified with Christianity rather than Samburu indigenous religion.

## Similar Knowledge Levels, Yet More Opposition among Formally Educated Samburu

To start to unpack whether “it is education alone, specific education on health and legal risks of FGM, or other confounding variables” (ICRW, 2016, p. 12) that influence the difference in FGM/C prevalence between “digital/modern” and “analogue/traditional” Samburu, people's knowledge of FGM/C health and other consequences was examined. Interlocutors were asked to list reasons why the government, NGOs, and others oppose FGM/C. All interlocutors were aware of the illegality of FGM/C in Kenya, and more than half could easily list the health risks of the practice. In fact, more “analogue” than “digital” Samburu could reproduce the common anticutting discourse often used by NGOs: FGM/C is harmful to the health of women and their babies, it takes away sexual pleasure, it is illegal and a human rights violation. “Analogue” interlocutors mentioned NGO workshops, NGO outreach officers who had visited their homesteads, and the radio as the sources of this information.

Despite the very similar levels of knowledge on FGM/C and its potential consequences, “digital/modern” Samburu more often said that they oppose FGM/C and would prefer the practice to end. Whereas some “digital” Samburu cited the health risks and legal implications of cutting as their reasons for opposing the cut, others said they did not have any specific reason other than the fact that the times are changing and cutting no longer fits their lifestyle aspirations. A group of male and female friends in their mid-20s who had attended university together explained to me that it was not formal education or information on the cut in itself that changed their mind, but rather the wider experience of living and studying in town and interacting with people from other ethnic groups that do not practice FGM/C. Some of them also mentioned that FGM/C goes against their Christian belief (see also Van Bavel, 2022). Some said that “cutting is something for those from the interior, those who are not educated.” Although many with a formal education agreed with the content of the anti-FGM discourse, opposing the cut has also become part of a “modern” identity and, to some extent, marks middle-class distinction.

Among “analogue” Samburu, knowledge of FGM/C consequences did not necessarily translate into opposition to FGM/C. Interlocutors said that, in response to anti-FGM legislation and NGO efforts, most “analogue” families did not abandon but merely altered the practice for it to be more easily hidden from law enforcers. As has been reported for most countries where FGM/C has been criminalized (Shell-Duncan et al., 2013), Kenya's Anti-FGM Act encouraged people to find a way of circumventing the law. Many Samburu families continue cutting in secret, often at an earlier age, and sometimes make a less invasive cut which is referred to as *Sunna* (see also Graamans et al., 2018, p. 80). Similar trends have also been found among other communities in Kenya (Kiage et al., 2014; Van Bavel, 2019) and in Tanzania (Van Bavel et al., 2017). Some “analogue” interlocutors who knew the anticutting rhetoric simply disagreed with it. A lay midwife in her 70s living close to Wamba said that in the three decades that she had helped women deliver, she had never seen the complications NGOs were warning them about. She had also assisted Turkana women (who do not practice FGM/C) and said she had not noticed any difference. Others did agree with the anticutting rhetoric that FGM/C is dangerous to women's health but considered the cut more important than the potential risk. One participant said that, to Samburu, “not being circumcised means you don’t exist, that you are actually dead” (female, 26 years old, higher education, Maralal). In a society where one is sanctioned with social death for not conforming, many people who would rather stop FGM/C—whether out of health, legal, or other concerns—continue the practice to avoid negative sanctions: “What is losing blood compared to losing my family, my community, my honor, my future?” (key informant, female, 30 years old, Form 2, Maralal).

Furthermore, FGM/C and its abandonment are profoundly political in Kenya. In the early 20th century, missionaries’ and colonial officials’ attempts to ban the practice resulted in large-scale resistance which further fueled the nationalist, anti-colonial movement (e.g., Njambi, 2007; Thomas, 2003). The first president of independence Kenya, Jomo Kenyatta, described female circumcision as “the very essence of an institution which has enormous educational, social, moral, and religious implications” (Kenyatta, 1965, p. 128). Kenya's second president, Daniel arap Moi, however, issued an official statement against female genital mutilation in 1982 after 14 girls died from FGM-related complications (Canada: Immigration and Refugee Board of Canada, 2000). More attempts to ban FGM/C in Kenya would follow, mostly to profound discontent among practicing communities, including their political representatives. In 1995, a female member of parliament tried to outlaw the practice, but her motion was defeated, reflecting parliamentarians’ reluctance to interfere with cultural practices, either out of fear of losing votes or because, as member of practicing communities, they valued the practice. In 2011, women parliamentarians from FGM/C-practicing communities succeeded in passing the Prohibition of FGM Act. The Act was received with “public protest, increasing medicalisation, greater secrecy as the practice moves underground, cutting girls at a younger age before they have the capacity to object, together with foot-dragging and non-compliance with the law” (Hughes, 2018, p. 279). In 2017, a female medical doctor petitioned against the 2011 Prohibition of FGM Act, calling the Act the “imposition of Western values.”

Similar sentiments were present among the Samburu interlocutors who participated in this study. A woman in her early 50s, without formal education and living in a rural area articulated it as follows:

They try to take away our culture and make us like Waswahili [Swahili for “Swahili people”]. They have tried that since many, many years. They try to make us farmers. They try to change our children in schools. And now they try to tell us that FGM/C is bad. [Interviewer: Who are they?] The government and wazungu [Swahili for “white people”].

Some interlocutors also showed some level of disdain for the “modern” Samburu who, in their eyes, abandoned Samburu culture. Two interlocutors mentioned that “modern” Samburu are no longer “real” Samburu. They described “real” Samburu as the Samburu who wear “traditional” Samburu clothes (including beaded jewelery), who primarily live from pastoralism, who practice Samburu indigenous religion, and who proudly follow all “traditional” rituals, including “female circumcision.” Scholars have placed this fear of cultural loss and resistance to anti-FGM/C efforts within a longer history of “outsider” anti-FGM/C efforts, political betrayal, land alienation, and marginalization of pastoralist groups in East Africa (Hodgson, 2001; Hughes, 2005; Van Bavel, 2019).

## Confounding Variables That Facilitate Abandonment for “Digital/Modern” Samburu

Although more “analogue/traditional” Samburu wanted “female circumcision” to continue, several said that they wanted FGM/C to end but found it impossible to abandon the practice. Two young mothers said that they did not want to cut their daughters because they had come to realize that the cut has no benefits and harms girls, but that they would likely do so anyway to ensure that their daughters would be respected and find a husband. A 32-year-old mother to three girls said after attending a workshop on the health risks of FGM/C: “We now know that it is dangerous for our girls. We are now willing to stop female circumcision. But how can we stop it if that means our daughters will be outcasts?” (female, 32-year-old, no formal education, married, village close to Wamba).

As Graamans and colleagues write (2018, p. 91), “legal prohibitions and awareness campaigns about sexual reproductive health and rights have met with limited success and might even be misguided [because] people still have other social concerns influencing their decisions, such as those related to marriageability, honor, and shame.”

Although “digitals” were more likely to oppose FGM/C, importantly, they also found it easier to abandon the practice because they would not be sanctioned in the same way as “analogue” women. One reason was that remaining uncut would not necessarily undermine “digital” women's marriage prospects. “Digital” women, especially those with formal education, are more likely to marry “digital” husbands who likely also oppose FGM/C and prefer to marry an uncut woman. Indeed, all young, formally educated men with whom I interacted expressed an explicit preference for a wife who was not cut. They expressed a desire for a “modern” monogamous, love-based marriage including sexual pleasure with their wife, which they believed to be more attainable with an uncut wife.

The alternative expectations with regard to marriage prospects were also reflected in the story of a 50-year-old father who had circumcised all but two of his daughters. I initially assumed that he had changed his mind about the practice over the course of his life and decided not to circumcise his youngest daughters, but then I learned that he only recently initiated his last-born daughter. When I asked him about it, he explained:

[The two uncircumcised girls] are the clever ones of the family. They did well in school. I knew they would finish form four and maybe even continue. They will find their husbands at school. Those boys from school don’t want their wives to be circumcised. (male, ± 55 years old, no formal education, village close to Wamba)

Similar to what Graamans et al. (2018, p. 90) have argued, the formal education of his daughters also helped him “save face, when [he] might otherwise be ridiculed by [his] peer for allowing [his] daughters not to undergo circumcision.” Interlocutors’ narratives showed that graduation from school is to some extent equated with graduation through circumcision and that higher levels of formal education shift the aspirations of young women as well as the social expectations that apply to them:

Girls who stay in the village grow up with our traditions. But those girls who go to school, they have their own way of graduating. And besides, when they have been to school, they no longer want to get circumcised. (male, 46 years old, no formal education, village close to Maralal)

My family doesn’t bother me about it because they know I went to school, so things are different for me. [Interviewer: How?] I am educated now so I don’t have to follow the rules from the village anymore. (female, 20 years old, higher education, rural Samburu Central)

Besides changing aspirations of “digital” Samburu and the different expectations that apply to them, the “digital” lifestyle—and, in particular, formal education—also make up for the loss of status that uncircumcised women experience. A 16-year-old young woman who dropped out of school after completing Form 1 explained:

My parents allowed me to stay uncircumcised because I was having good grades at school. But when I was supposed to go to Form 2, we lost our cows and we no longer had money to pay for my school fees. Then my parents decided that I should be circumcised. [Interviewer: How did you respond?] I agreed because without education and without circumcision, how would I be respected? (female, 16 years old, Form 1, unmarried, Baragoi)

As her case shows, her formal education could substitute for the status she would otherwise gain from being cut. However, if the level of formal education attained is considered too low to substitute for the “traditional” graduation including circumcision, girls will still be cut to ensure the status and respect of the girl and her family.

Graamans et al. (2019, p. 6) write that “the assumption behind increased education is that women who are educated are aware of their rights and will be able to better claim and defend these rights for themselves and their daughters/children.” My findings confirm that formal education comes with “modern” aspirations and a better ability to stand up for their rights. In addition, however, the narratives of my interlocutors suggested that it was also the alternative expectations that applied to them and the fact that formal education replaced FGM/C as providing them with a respectable status that allowed them to reject the cut.

In summary, while some “analogue” Samburu wanted FGM/C to end, they faced more barriers to effectively abandoning the cut than “digital” Samburu. This is in line with evidence in Kuria and Kisii where the “isolation and stigma directed towards uncircumcised girls and women [was found to be] far greater for uneducated girls than for those who are educated” (Oloo et al., 2011, p. 4).

## Circumventing Cutting Expectations

Families and individuals without formal education found it harder to publicly denounce FGM/C, because of the sanctions that would follow. However, some found ways of circumventing the social expectation by “staging” the cut. I found this in particular among Samburu living close to the urban center of Maralal. Interlocutors said that FGM/C is coming to an end in Maralal, on one hand, due to the higher educational and literacy levels (KDHS, 2014, p. 42), but also because closer to town it is harder to hide the illegal practice from the police. However, as in other parts of Samburu County, some families continue cutting in secret. This secrecy creates an atmosphere of uncertainty around the cutting status of women. Some Samburu without formal education who do not want to cut their daughters use this atmosphere of secrecy and uncertainty to pretend that they already cut their daughter in secret at night to hide from the police while they actually have not and are not intending to do so.

Due to legal and ethical concerns, I never asked women about their cutting status. However, a woman living in a rural area close to Maralal entrusted to my interpreter and me that she had not been circumcised and that she was unsure whether her husband knew. We had interviewed her husband in town a few days before, and he had said:

I wanted to marry an uncircumcised woman. But it can be a challenge because the parents decide. Now I am not sure whether my own wife is circumcised or not—I don’t dare to tell her to show me. But if it turns out that she isn’t, and they would want to take her to get circumcised, I would refuse, because I like her uncircumcised. (Lmurani, 25 years old, no formal education, village close to Maralal)

His wife's family found a way of avoiding the repercussions of not cutting by using the ambiguity and secrecy resulting from FGM/C being illegal to pretend to cut.

Some interlocutors also mentioned that economic capital could help in pretending that a girl is cut. Leshan, the motorcycle-taxi driver whose family is not formally educated and lives in a more interior area of Samburu East, explained: “I will just give the women something small so that they do not have to check my bride and can pretend that she is circumcised. Even the bride herself, she can give them something so that they can lie” (Lmurani, 28 years old, Form 2, unmarried, Samburu East).

In Samburu West, a recently married man and his best man told me the story of how they went to see the bride's family a few days before the wedding to convince them not to circumcise her. The father, however, refused, stating that his daughter is a respectable Samburu woman who would undergo the cut just like her ancestors. Feeling defeated, the men wanted to leave the family's compound when the mother of the girl called them for a cup of tea. In the secrecy of her house, she told them: “If you can give us something for tea and sugar, and something we can give to our husband to flatter him (*kumbembeleza*, Swahili), then we [the women] can lie to him and say she's cut. She’ll come to you just like she is now.” The men did as she suggested, and a few days later the groom married an uncut wife. Economic capital allows for bribing family members to deceive those who insist on FGM/C to think that the woman in question has been cut when she has not.

A key informant explained that economic capital also provides more freedom to deviate from society's expectations because having economic resources makes one less dependent on the rest of society.

If you are not depending on the community for your survival, it is much easier to cope with the pressure and stigma that comes with choosing not to practice circumcision. People cannot threaten to take away their support, because you support yourself. And marriage will still be possible because people are more likely to accept an uncircumcised woman if the marriage links them to a prosperous family. (key informant, male, 25 years old, Maralal)

Graamans et al. (2019, p. 6) suggest that economic resources may “reduce [women's] dependency and give them more economic stability, as women have their own income.” Indeed, a 27-year-old woman who was among the “pioneers” who refused being cut remembered that it was very hard at first to refuse to be cut. With the support of a microcredit program, she started a little shop in second-hand clothes in Maralal. She told me: “I no longer depend on my father or uncles. […] Now they sometimes come to lend money from me. So they no longer criticize me. They respect me because they need me and because they see my success.”

In summary, formally educated Samburu more often opposed FGM/C and found it easier to effectively abandon the practice because their formal education replaced FGM/C as a source of status and respect. Those Samburu without formal education who opposed FGM/C—often, these Samburu lived closer to town and economic activities besides pastoralism and agriculture—found it harder to publicly denounce the practice for fear of losing their status and being ostracized. However, the secrecy around the practice resulting from anti-FGM legislation created a gray zone that families can use to pretend to cut their daughters. Furthermore, financial resources can help people bribe others to pretend to cut and/or make people less financially dependent on others and therefore freer to choose one's own path, despite potential ridicule and exclusion.

## Interrogating the “Ignorant Pastoralist” Narrative

My findings show that formally educated Samburu did not necessarily have more knowledge on the health and legal implications of FGM/C. Upon asking about FGM/C's health risks, a 25-year-old formally educated man said: “I don’t know, I just know they say it's not good so that's why I’m against it.” For many “analogue” Samburu, the knowledge of the health and legal implications of FGM/C did not necessarily translate into abandoning the cut because of the different expectations that rest on them and fewer alternative options available to them. These findings challenge the assumption, at times implicitly present in anticutting rhetoric, that Samburu continue FGM/C out of “ignorance.” Although more research is necessary, my findings also start to show that the link between formal education and the abandonment of FGM/C among Samburu is less due to the levels of knowledge related to FGM/C, and more due to the fact that formal education can replace FGM/C as a source of status and respect and provides people with a new socioeconomic position to which the expectation of cutting does not (or, at least, less strongly) apply.

Yet, the assumption of ignorant pastoralists continues to seep through in some of the anticutting efforts. An intern working at a development organization targeting child marriage and FGM/C in Samburu said: “We try to enlighten them about the health effects, but they are just stubborn. They love this culture so much. They refuse to change, even when they know how it can harm their girls”(female, 26 years old, NGO staff, non-Samburu).

One of my key informants suggested that a century of having been treated as “ignorant” and backward, first by the colonial and later by the post-Independence government, has impacted the way Samburu see themselves and their position as passive recipients of “development.” I witnessed an awareness-raising session on FGM/C led by NGO staff members from communities that do not practice FGM/C. After the workshop, I overheard a group of Samburu women complain that the facilitators had talked about infibulation, seemingly unaware that it is not the type that Samburu practice. When I asked why no one had told the facilitators that Samburu don’t perform infibulation, Nashami, a woman in her mid-30s, said: “They are the experts. They are learned and from the city. We are just Samburu women. We didn’t even go to school. What do *we* know? We only know our village. We are just grateful that they come to help us.”

On another occasion, a Samburu midwife told me how an NGO had paid her and other midwives to “drop their knives,” assuming that FGM would end when circumcisers stop cutting. Her daughter had gone to Nairobi to study to be a medical doctor, and under her influence the midwife had slowly changed her attitude on FGM/C, hoping that, with time, the practice would come to an end. When the NGO had come to her region, she had felt hopeful that they would speed up the process. As soon as the project manager explained their approach, however, she had felt disappointed because she knew it would not work. “Parents would just find midwives from other places to come cut their daughters, or some of us would take the money and still cut secretly.” The project went ahead and, as predicted by the midwife, failed to discourage people from practicing female circumcision. She too had remained silent when they presented the project, lacking the confidence to recognize her own insights as valuable: “They came with their plans from Nairobi. They never asked us. They are the experts. I thought they knew more than we do.”

These are only two examples of the many instances that I witnessed where men and women from practicing communities listen to the so-called “experts” making wrong statements about female genital cutting or their cultures in general or bringing in projects that were meant to fail from the outset. Sometimes, when I asked people about certain ceremonies or rituals, they would apologetically say that they had not gone to school so did not know the answer, even though they grew up attending those ceremonies every few months. They felt that their way of knowing or understanding their community and life was not the “right” way and would often point me to formally educated people or, on a few occasions, a US researcher active in the area, for the “right” answers to my questions.

The ignorant pastoralist trope is not the invention of anticutting activists, however. Discourses that portray Kenyan pastoralists as traditionalist, backward, uneducated, and in need of “enlightenment” date back to the colonial period when the British colonial government started its process of continuous land alienation and sociopolitical marginalization of pastoralist groups (Hughes, 2006; Straight, 2020). Portraying pastoralists as ignorant and incapable of taking care of the land justified the continuous land alienation, infantilization, and sociopolitical marginalization of pastoralists for colonial and settler interests (Hughes, 2006; Straight, 2020). These policies and the stereotypes underpinning them were continued by the Independent government, which formalized the unequal allocation of resources to the agricultural sector (Catley et al., 2013, p. 3).

Hodgson (2001) has shown for Tanzanian Maasai how, throughout history, they became understood and portrayed as stubborn, backward pastoralists, not only by the colonial and independent governments but also by development workers. “For certain […] Africans,” Hodgson (2001, p. 3) writes, “‘The Maasai’ represent embarrassing reminders of a lifestyle now despised and denigrated.” The same discourse is used in government and donor meetings and inspires many of the projects aiming to “develop” pastoralists (Catley et al., 2013, p. 6). In her research among Maasai in northern Tanzania, Bishop (2007, p. 19) found that “many of those Maasai who went to school are keen to distance themselves from the image of backward pastoralist that they encountered at school and in multi-ethnic environments surrounding schools.” Maasai have also often been called “backward” or “stubborn traditionalists” in response to their perceived resistance to development projects trying to end FGM/C or bring other cultural change (Bishop, 2007; Winterbottom et al., 2009). The narratives of anticutting activists in Samburu with whom I spoke reflected similar assumptions and put the blame for not achieving the envisioned results on the conservatism and ignorance of Samburu. As the above vignettes show, some Samburu have come to internalize these discourses and value their own knowledge less than that of “outsider experts.” Francis, a young university-educated Samburu man, described how he felt inferior to his non-pastoralist peers at university in Nairobi:

They would call us [pastoralists] ndawuo, which means a person who doesn’t know much, someone you can easily fool, someone who is naive and simple. I started to dress differently, just to not look like a ndawuo. It is a really humiliating and demeaning term. When people talk about pastoralists like that, what does that do to one’s self-esteem? If that is how they see us and how they talk about us, how will we start seeing ourselves? As stupid? Incapable? It makes us wait for them to come help us, because we think we can’t do it. (male, 25 years old, higher education, Maralal)

As Francis suggested, and as these examples seem to confirm, stereotypes about ignorant pastoralists that have proved persistent throughout the century harm pastoralist communities: indirectly when they underpin well-intended development interventions that do not acknowledge the insights of people and hence produce unintended consequences, and directly through the discursive violence they inflict on pastoralists, harming their self-image and worth. In addition, narratives that blame the “stubbornness” of Samburu for continuing FGM/C despite having been “educated” on its health and legal implications, overlook the sociocultural significance the practice holds within society, and the social pressure individuals and families experience to comply with the norm. Most importantly, it overlooks the structural aspect of lower access to formal education and higher poverty rates that creates little alternative opportunities for Samburu, shifting the blame away from the government's failure to provide equal access for all Kenyan communities toward a community stereotyped as backward, stubborn, and therefore guilty. As my findings show, the difference between those who abandoned the cut and those who did not lay not so much in the difference in knowledge, but rather in the alternative possibilities that come with formal education and a “digital,” “modern,” or “middle-class” identity more generally. Those with formal education or financial resources can either abandon the cut without repercussions or find ways of circumventing the repercussions that normally come with not cutting. Those living closer to urban centers used the atmosphere of secrecy due to the criminalization of FGM/C to their advantage.

Anthropologists and anticolonial scholars have long criticized “Western” anti-FGM discourses for reducing African women to the passive victims of their backward cultures and patriarchal men (Mohanty, 1988; Nnaemeka, 2005). My findings add to these critiques that anticutting rhetoric in Kenya also reinforces and reproduces the trope of the ignorant pastoralist. Such rhetoric is not only harmful because of the violence it inflicts on the self-esteem of a community but, as Straight (2020) argues, it also contributes to a century-old discourse that blames pastoralists—their culture, their backwardness, low education level, their stubbornness, and so on—for their situation, thus distracting from a long history of land alienation and sociopolitical marginalization in which the colonial and independent governments played an important role. Throughout the last century, the trope of the ignorant pastoralist has also legitimized “land policies that favor farmers, settler descendants, and other Kenyan elites” (Straight, 2020, p. 15). The actions of anticutting activists and organizations can have far-reaching material and epistemological consequences. It is therefore important to challenge the assumption that the continuation of FGM/C is due to the ignorance of Samburu, which can be solved by “awareness raising” sessions alone, which some anticutting activists whom I interviewed described as “bringing enlightenment to the ignorant.”

## Conclusion

Research has shown that there is a correlation between educational attainment and abandonment of FGM/C. However, there is a lack of evidence on what explains this correlation. Some of the anticutting activists and professionals I interacted with in Samburu County and wider Kenya sometimes blamed the assumed “ignorance” of Samburu, allegedly reflected in low literacy and educational levels, for the slow decrease of FGM/C prevalence in Samburu County. My findings, however, challenge the assumption that it is “ignorance” of FGM/C health and legal implications that makes Samburu continue cutting. Formally educated Samburu did not necessarily stop cutting because of the health risks or illegality of FGM/C; rather, for women, formal education provided them with status and respect, therefore releasing them and their families of the expectation to be cut. In general, formally educated Samburu aspired to a “modern” lifestyle and were not expected to adhere to the custom of “female circumcision.” Among “analogue” Samburu, a significant number was aware of the health and legal implications of FGM/C. Although many of them wanted to continue the cut regardless, others wanted to abandon it yet felt that the repercussions for doing so were too severe. For those living in the urban area of Maralal, where the cut has gone underground due to its criminalization, some “analogue” Samburu were able to use this secrecy to pretend to cut. Others used their economic capital to buy the silence or complicity of family members in pretending that a daughter or bride was cut. Notably, gender did not predict people's attitudes toward FGM/C; interlocutors’ educational level, rural or urban livelihood, and socioeconomic status played a far more important role.

Detangling the new social expectations and possibilities that come with formal education from the direct cause-and-effect of “knowledge” on FGM/C abandonment is important because it challenges the notion that “the problem” of the slow decrease of FGM/C among Samburu lies with “ignorance.” This notion, as I showed, has its origins in colonial times and continues to inform much of the government and development policies directed at pastoralists. Besides the unintended consequences of projects influenced by these assumptions, these stereotypes also inflict discursive violence on Samburu. Furthermore, in putting the blame on the ignorance of Samburu, the discourse overlooks the structural inequality and long history of marginalization of pastoralists that have led to higher illiteracy and lower educational levels among Samburu, which in turn generate fewer opportunities to choose not to continue FGM/C.

These findings should not be misread or used to minimize the importance of sensitization and awareness-raising campaigns; community sensitization on health and human rights has played a critical role in raising awareness and empowering women to claim and defend their rights. My argument is about the importance of being conscious of unintended messages sometimes implicitly present in anti-FGM rhetoric. A conscious, nuanced rhetoric acknowledges that “knowledge” might change people's attitudes on FGM/C but might not be enough for people to change their behavior. As has been argued by others and has been put into practice by NGOs across Kenya and beyond (e.g., Diop & Askew, 2009; Van Bavel, 2019), awareness raising should be accompanied by creating an environment in which FGM/C abandonment does not result in ridicule and ostracism. In West Africa, for example, the Senegal-based NGO Tostan encourages villagers to organize a public declaration at the end of their three-year nonformal education program in which the community commits to embracing uncut women. Among the Loita Maasai of southern Kenya, the NGO SAFE Maa has worked with the cultural leaders to officially bless an alternative rite of passage that omits the cut, which means that customary law prohibits excluding women who were initiated through this alternative rite. Similar approaches could be explored in Samburu. Anticutting efforts will also need to take into account the political nature of FGM/C in Kenya, and the fact that some Samburu experience anticutting efforts as an attack on their culture and threat to the existence of their ethnic group. Most importantly, the findings show the crucial role of formal education in bringing social change and emphasize the importance of ensuring all Samburu can access schools.

## Notes

1. Lmaa for a gathering of clan members in a temporary shelter usually built for cultural festivals. The gathering can last for a few months. Lorora settlements are more common now, especially in Samburu North, because they offer protection in times of insecurity.

2. Samburu use “Swahili” to refer to Kenyan ethnic groups that have adopted, what they consider to be, a more “Westernized” lifestyle, including clothing.

3. All names are pseudonyms.
